# 965. Advanced Practice Providers in Infectious Disease: Educational Needs and Opportunities

**DOI:** 10.1093/ofid/ofab466.1160

**Published:** 2021-12-04

**Authors:** Leah H Yoke, Alison M Beieler, Catherine Liu, Steven A Pergam, Steven A Pergam, Shireesha Dhanireddy

**Affiliations:** 1 University of Washington; Fred Hutch Cancer Research Center, Seattle, Washington; 2 Harborview Medical Center, Seattle, Washington; 3 Fred Hutchinson Cancer Research Center; University of Washington, Seattle, Washington; 4 University of Washington, Seattle, Washington

## Abstract

**Background:**

Advanced Practice Providers (APPs) practice throughout Infectious Disease (ID) in a variety of settings through interprofessional collaboration with physicians, pharmacists, and other team members. However, there is a paucity of specific and directed educational opportunities available for APPs within ID. In order to better understand this, we examined specific APP educational needs and how educational programs could provide high quality opportunities for APPs in ID.

**Methods:**

Voluntary anonymous surveys were created in the REDCap data tool and distributed by email lists, social media, and Infectious Diseases Society of America community forums to APPs working in ID.

**Results:**

Ninety-nine APPs responded to the survey (figure 1). 97% (96) of respondents were interested in APP specific ID educational opportunities. Of respondents, 76% (74) felt ID specific podcasts would be most helpful, while 86% (84) noted that access to ID clinical case conferences or self-directed, online modules would be instructive (figure 2). 91% (90) did not attend IDWeek annually due to various barriers, including lack of clinical coverage and cost associated with the conference (figure 3) despite 89% (88) receiving Continuing Education (CE) reimbursement. 64% (62) respondents were interested in future APP mentorship opportunities, from either more senior APPs or physicians.

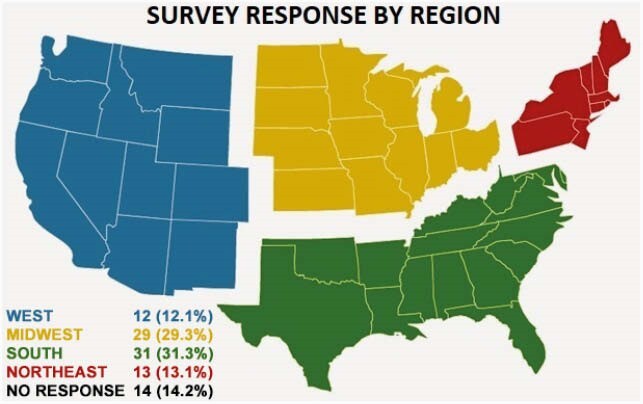

Figure 1. Geographic Distribution of Respondents, n=99

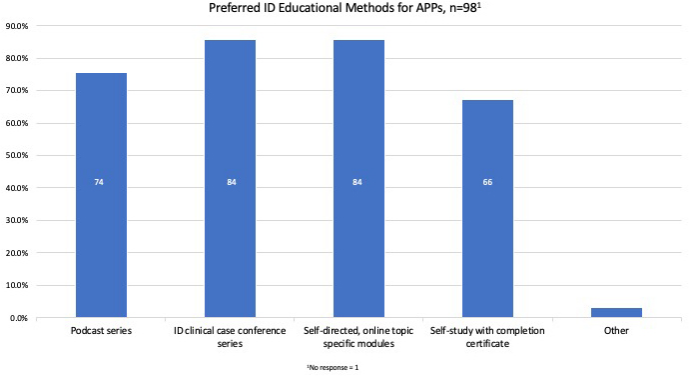

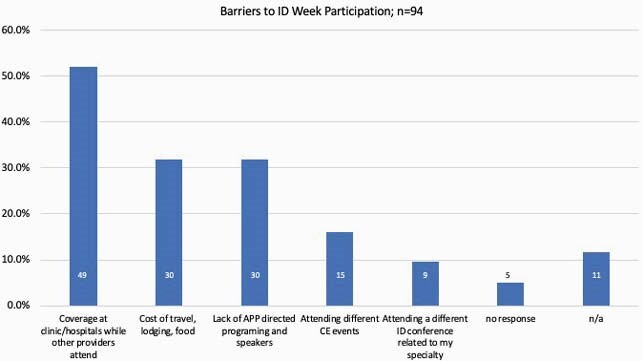

**Conclusion:**

APPs provide collaborative and specialized ID care in a variety of settings. However, continued educational needs specifically for APPs have been identified. From survey respondents, the majority of APPs did not attend IDWeek, a sentinel ID education event, citing clinical coverage and cost being significant barriers. This represents an opportunity for clinically focused educational opportunities, both at IDWeek and also through other platforms, particularly since many APPs receive CE funding from their employers. Podcasts, online lecture series, and self-study certificate programs were identified as avenues for ID teaching and also present accessible, alternative methods for training. Ultimately, as the growing APP workforce continues to provide patient care in a variety of ID settings, educational opportunities with mentorship are necessary to support them in their practice.

**Disclosures:**

**Steven A. Pergam, MD, MPH**, **Chimerix Inc.** (Other Financial or Material Support, Clinical Trial)**Global Life Technologies, Inc.** (Grant/Research Support)**Merck and Co.** (Other Financial or Material Support, Clinical Trial) **Steven A. Pergam, MD, MPH**, Chimerix (Individual(s) Involved: Self): Clinical Trial; Global Life Technologies, Inc (Individual(s) Involved: Self): Research Grant or Support; Merck & Co. (Individual(s) Involved: Self): Scientific Research Study Investigator; Sanofi Aventis (Individual(s) Involved: Self): Other Financial or Material Support, Provided vaccines for clinical trial sponsored by the NIH

